# Sorption of ammonium and nitrate to biochars is electrostatic and pH-dependent

**DOI:** 10.1038/s41598-018-35534-w

**Published:** 2018-12-04

**Authors:** Rivka B. Fidel, David A. Laird, Kurt A. Spokas

**Affiliations:** 1 Department of Soil, Water and Environmental Science, The University of Arizona, Tucson, USA; 2 Department of Agronomy, Iowa State University, Ames, USA; 3 USDA-ARS, Soil and Water Management Unit, University of Minnesota, Minneapolis, USA

## Abstract

Biochars are potentially effective sorbents for NH_4_^+^ and NO_3_^−^ in water treatment and soil applications. Here we compare NH_4_^+^ and NO_3_^−^ sorption rates to acid-washed biochars produced from red oak (*Quercus rubra*) and corn stover (*Zea mays*) at three pyrolysis temperatures (400, 500 and 600 °C) and a range of solution pHs (3.5–7.5). Additionally, we examined sorption mechanisms by quantification of NH_4_^+^ and NO_3_^−^ sorption, as well as Ca^2+^ and Cl^−^ displacement for corn stover biochars. Solution pH curves showed that NH_4_^+^ sorption was maximized (0.7–0.8 mg N g^−1^) with low pyrolysis temperature (400 °C) biochar at near neutral pH (7.0–7.5), whereas NO_3_^−^ sorption was maximized (1.4–1.5 mg N g^−1^) with high pyrolysis temperatures (600 °C) and low pH (3.5–4). The Langmuir (r^2^ = 0.90–1.00) and Freundlich (r^2^ = 0.81–0.97) models were good predictors for both NH_4_^+^ (pH 7) and NO_3_^−^ (pH 3.7) sorption isotherms. Lastly, NH_4_^+^ and NO_3_^−^ displaced Ca^2+^ and Cl^−^, respectively, from previously CaCl_2_-saturated corn stover biochars. Results from the pH curves, Langmuir isotherms, and cation displacement curves all support the predominance of ion exchange mechanisms. Our results demonstrate the importance of solution pH and chemical composition in influencing NH_4_^+^ and NO_3_^−^ sorption capacities of biochar.

## Introduction

Anthropogenic perturbations to the nitrogen cycle have resulted in elevated concentrations of inorganic N in natural water bodies, threatening human health and aquatic ecosystems. Excess N predominately originates from nitrogen fertilizers, animal manures, municipal waste, and chemical processing facilities – which either add NH_4_^+^ and NO_3_^−^ to soil or water directly, or contribute to their formation through biochemical mineralization and/or redox transformations. Furthermore, the addition of N to the environment from fertilizers and anthropogenic wastes indirectly exacerbates climate change by contributing to N_2_O emissions^1,2^. Most available technologies for removing ammonium (NH_4_^+^) and nitrate (NO_3_^−^) from waters, such as membrane filtration and biological and chemical treatments are expensive and/or inefficient at removing N^3–5^.

Biochar produced from the pyrolysis of biomass feedstocks has the potential to be a low cost and efficient sorbent for NH_4_^+^ and NO_3_^−^, through both soil applications and use in water treatment^6–8^. As a soil amendment, biochar may reduce NO_3_^−^ leaching and improve nitrogen use efficiency, which can increase crop yields^9,10^. Furthermore, pyrolysis of biomass to produce bioenergy and biochar co-products is carbon-negative when appropriate feedstocks and pyrolysis techniques are used, and hence use of biochar as a sorbent and soil amendment can help mitigate climate change^11–13^.

A wide range in efficacy of different types of biochar for removal of NH_4_^+^ or NO_3_^−^ from various aqueous solutions has been observed^7,8,14^. When co-composted with plant materials and manures, biochar has been shown to enhance NO_3_^−^ retention of compost and subsequently increase plant growth following application^15^. Some biochars decrease NH_4_^+^ and/or NO_3_^−^ leaching from soils under specific contexts (i.e., fertilizer type, soil type, and leaching conditions), but these effects have been inconsistent across studies employing different biochars and soils^9,16–22^. Such inconsistencies suggest underlying mechanisms which are context-sensitive. Biochar’s capacity to sorb NH_4_^+^ and NO_3_^−^ is often attributed to its physical (high surface area and porosity) and chemical (negatively and positively charged functional groups) properties, respectively^21^; however evidence of mechanistic connections between biochar properties is lacking and there is no consensus on if or when one mechanism can dominate over the rest. Thus, more fundamental research investigating mechanisms underlying NH_4_^+^ and NO_3_^−^ sorption to biochars is needed in order to develop protocols for the production and use of biochar as an inorganic N sorbent.

Sorption of NH_4_^+^ by biochars produced from diverse lignocellulosic feedstocks at a wide range of temperatures has been previously quantified using batch equilibration procedures^23–29^. Ammonium sorption capacity consistently increases with decreasing pyrolysis temperature for comparisons of biochars produced from a common feedstock. However, comparing between studies, biochars produced from similar feedstocks at similar temperatures do not always exhibit similar sorption behavior. For example, Wang *et al*.^23^ report 0.86 and 0.58 mg g^−1^ of NH_4_^+^-N sorption by maple hardwood biochar produced at 300 °C and 400 °C, respectively using a 80 mL:1 g solution:biochar ratio; however, Hollister *et al*.^28^ report a sorption capacity of 0.01 mg g^−1^ NH_4_^+^-N for an oak biochar produced at 350 °C using a 100 mL:1 g solution:biochar ratio (both biochars were produced by slow pyrolysis, and sorption was quantified from solutions containing 100 mg-N L^−1^). Gai *et al*.^26^ and Yang *et al*.^25^ investigated NH_4_^+^-N sorption on slow pyrolysis wheat straw biochars produced at 400–700 °C, and 350 & 550 °C, respectively – but reported substantially different model parameters for Langmuir and Freundlich isotherms. However, from these studies we can compile some interesting insights into sorption mechanisms. Yang *et al*.^25^ examined the effects of solution pH and cation competition, and found that NH_4_^+^ sorption increased with increasing pH for all three biochars studied, but decreased when additional base cations were introduced^25^. Likewise, Wang *et al*. (2015 and 2016) observed increased NH_4_^+^ sorption to biochars after adjusting the solution pH to 7^23,24^. Some of the inconsistencies in the literature may be attributed to differences in the concentration of soluble base cations found in fresh (unwashed) biochars, which may compete with NH_4_^+^ for sorption sites. Additionally, most biochars contain soluble alkalis^30^, which can influence solution pH during sorption measurements. Gai *et al*.^26^ attempted removal of interfering compounds by acid washing three of twelve analyzed biochars. In that experiment, wheat straw, peanut shell, and corn stover biochars produced at 500 °C sorbed 0.63, 0.73, and 2.12 mg NH_4_^+^-N g^−1^ before acid washing, respectively, and 0.27, 0.45 and 0.43 mg NH_4_^+^-N g^−1^, respectively, after acid washing^26^. Hence, acid washing decreased NH_4_^+^ removal; however, the final solution pHs were not reported and the effect of biochar pyrolysis temperature on sorption to acid-washed biochars was not investigated. Thus, an overall trend for increased NH_4_^+^ sorption with increasing solution pH and decreasing biochar pyrolysis temperature has emerged in the literature. However, no single study has systematically examined the effects of biochar feedstock and pyrolysis temperature on sorption, while controlling for solution pH and competing cations.

Although several studies have analyzed for NO_3_^−^ sorption to biochars^4,26–28,31^, few studies report significant sorption without chemical modification^4,26^. Chintala *et al*. (2013) report up to 0.1 mg g^−1^ of NO_3_^−^-N sorption to untreated fast pyrolysis biochars, as well as linearly decreasing sorption rates with increasing solution pH, and decreased sorption in the presence of phosphate and sulfate. Gai *et al*.^26^ were unable to detect NO_3_^−^ sorption to untreated biochars, but after washing with water and acid, sorption increased to 0.021–0.032 and 0.037–0.058 mg g^−1^ of NO_3_^−^-N, respectively. Neither study examined the effect of pyrolysis temperature on NO_3_^−^ sorption to acid-washed biochar. Together Chintala *et al*. (2013) and Gai *et al*.^26^ provide evidence for both pH and competitive anion effects on NO_3_^−^ sorption to biochar. Neither study considered the effects of soluble anions or alkalis found in the biochar on NO_3_^−^ sorption. Thus, no studies have systematically controlled both pH and competitive anion effects in investigating NO_3_^−^ sorption by biochars.

Here we aim to investigate the effects of aqueous solution properties (pH and competitive ion concentrations) and of biochar production parameters (feedstock and pyrolysis temperature) on NH_4_^+^ and NO_3_^−^ sorption. We hypothesize that, when controlling for differences in native soluble alkalis and ions found in biochar, (1) both biochar feedstock and pyrolysis temperature will influence NH_4_^+^ and NO_3_^−^ sorption, (2) increasing solution pH will increase NH_4_^+^ sorption and decrease NO_3_^−^ sorption due to ion competition (with H^+^ and OH^−^, respectively) and/or changes in surface charge, and (3) both NH_4_^+^ and NO_3_^−^ sorption to biochar occur through competitive electrostatic ion exchange mechanisms.

## Methods

### Biochar production

Red oak (*Quercus rubra*) and corn stover *(Zea mays)* feedstocks were slow-pyrolyzed at 400, 500 and 600 °C to produce a total of six biochars. Slow pyrolysis was conducted in a N_2_-purged muffle furnace (Thermo-Scientific; Lindberg/Blue M Moldatherm box furnace)^32^. About 500 g of each air-dry feedstock (particle size < 2 mm) was placed into a steel box (24 cm × 14 cm × 15 cm) within the muffle furnace; an N_2_ purge line was inserted through the furnace and into the box, and the box was purged for 30 min at 1500 mL min^−1^. For the pyrolysis reaction, the purge rate was decreased to 40 mL min^−1^, the furnace was initially heated at 10 °C min^−1^ to a temperature 100 °C below the target temperature (300 °C, 400 °C or 500 °C), held for 2 hr, and then heated to the final temperature (e.g., 400, 500 and 600 °C) at 0.5 °C min^−1 ^^32^. The highest treatment temperature was held for 2 hr, then the furnace was allowed to cool overnight under N_2_-purge (40 mL min^−1^). Following cooling, all biochars were ground and sieved to <0.5 mm.

### Acid washing and elemental analysis

All biochars were acid-washed to remove soluble salts and alkalis that could interfere with sorption analysis^4,30,33^. Briefly, biochars were first acid washed by equilibrating with 0.05 M HCl for 24 h to dissolve and remove soluble salts and alkalis. Subsequently, the biochars were washed for 15 min each with 1 M CaCl_2_ twice (to ensure homogeneous ion saturation), then four times with ultra-pure deionized water (18 MΩ) to remove excess salts. All washes were conducted in centrifuge tubes on a reciprocating shaker using a 50 mL:1 g ratio of solution:biochar, and vacuum filtration (0.45 µm nitrocellulose filter paper) to remove the aqueous phase between wash steps. After the final wash, biochars were dried at 65 °C for 72 h.

A preliminary test was conducted to confirm the efficacy of this acid washing procedure by analyzing the extracts of the 500 °C corn stover biochar conserved from each washing step (see ion displacement section below). The 500 °C corn stover biochar was chosen because it was produced at the median pyrolysis temperature (for this study), and previous research showed that corn stover biochar usually has more soluble salts than wood biochar^30^. Results showed that the initial HCl extract contained 14.2 mmol L^−1^ base cations in total and 0.7 mmol L^−1^ other elements (P, S, Fe, Al, Cu and Zn), whereas the extract of the fourth water wash contained 0.058 and 0.116 mmol L^−1^ of Ca and Na, respectively, and <0.02 mmol L^−1^ total of all other analyzed elements (K, Mg, P, S, Fe, Al, Cu and Zn). The efficacy of this washing procedure is also supported by previous evidence^30,33^. Henceforth these biochars will be referred to using an abbreviation consisting of letters representing their feedstock (RO and CS for red oak and corn stover, respectively), followed by a number representing the first digit of their peak pyrolysis temperature (4, 5 and 6 for 400, 500 and 600 °C, respectively), and finally a “s” denoting “slow pyrolysis.” Total percent C, N, H and S of the acid-washed biochars were determined by combustion analysis (Vario Microcube, Elementar).

### Batch sorption experiment with varying solution pH

To determine the effect of solution pH, biochar feedstock, and biochar pyrolysis temperature on NH_4_^+^ and NO_3_^−^ sorption to biochar, a batch sorption experiment was conducted. For each of the six acid-washed biochars, 0.1 g was weighed into 40 mL centrifuge tubes in duplicate with 25 mL of solution containing 10 ppm each of NH_4_^+^-N and NO_3_^−^-N, and a variable amount of Ca(OH)_2_. To make each solution, 10–20 mL of water was pipetted into to the tube, then 0–10 mL of standardized 1 mmol L^−1^ Ca(OH)_2_, and finally 5 mL of 3.75 mmol L^−1^ NH_4_NO_3_ solution (50 ppm each of NH_4_^+^-N and NO_3_^−^-N). Adding the solutions in this order minimized the risk of NH_3_ volatilization by allowing the acidic biochars to react with the Ca(OH)_2_ prior to the addition of NH_4_^+^. The samples were equilibrated on a reciprocating shaker for 24 hr^25^ and allowed to settle for 20–40 min. The sample supernatants were analyzed for pH, then syringe filtered to <0.45 µm (nitrocellulose filter paper, VWR). The addition of Ca(OH)_2_ resulted in sample supernatant pHs ranging from ~3.5 to 9. However, only samples with pH < 7.5 were used to avoid the potentially confounding effects of NH_3_ volatilization. Filtrates were either analyzed for NH_4_^+^ and NO_3_^−^ the same day or frozen until analysis. To measure NH_4_^+^ and NO_3_^−^ concentrations, all filtrates were prepared with the Berthelot and Griess-Ilosvay reagents, respectively, and analyzed colorimetrically using a microplate reader^34^. Preliminary results showed that absorbances of controls, water-only biochar extracts without NH_4_NO_3_ addition both with and without the reagents added, were comparable with distilled water. Each sorption curve (N sorbed vs. pH) was fit with linear, exponential, and power models in Excel; the models with the highest r^2^ values were presented.

### Batch sorption with varying N concentrations

A batch sorption experiment was conducted to construct NH_4_^+^ and NO_3_^−^ sorption isotherms for the corn stover biochars. Using results from the pH experiment described above optimum conditions were identified, including the sorption pHs for each analyte and the amount of Ca(OH)_2_ (1 mmol L^−1^ solution) required to bring each biochar to that pH. Each of the three corn stover biochars (0.1 g) was weighed into 50 mL centrifuge tubes. For each analyte, appropriate amounts of Ca(OH)_2_ (7–8.1 mL for NH_4_^+^; 0 mL for NO_3_^−^) and water were added to each biochar sample, along with 1.25–10 mL of 3.75 mmol L^−1^ NH_4_NO_3_ solution (21 samples for each biochar-analyte combination; 126 samples total). Samples were equilibrated, analyzed for pH, filtered, and then analyzed for NH_4_^+^ and NO_3_^−^ as described above. When a sample was found to have a final pH differing from the desired pH by more than 0.25 pH units, the sample was discarded and a replacement sample was prepared from the same biochar. The resulting isotherms were fit using both Freundlich and Langmuir models^26,35^.

### Ion displacement

To assess whether NH_4_^+^ and NO_3_^−^ sorption to biochars occurs through an electrostatic adsorption mechanism, filtrates from two selected biochars (previously acid-washed and CaCl_2_-saturated, see above) were conserved from the sorption experiment described in the preceding paragraph, and analyzed for ions displaced from the biochar. For NH_4_^+^, the corn stover biochar that sorbed the most NH_4_^+^ was chosen, and filtrates were analyzed for Ca^2+^ by inductively-coupled plasma optical emission spectroscopy (Thermo Scientific™ iCAP™ 7400 ICP-OES Analyzer). For NO_3_^−^, the corn stover biochar that sorbed the most NO_3_^−^ was chosen, and filtrates were analyzed for Cl^−^ colorimetrically (Seal Analytical method #EPA-105-A Rev. 5; equivalent to US EPA method 325.2). These two biochars were chosen to ensure detection of ion displacement and maximize accuracy. Net ion displacement associated with NH_4_^+^ and NO_3_^−^ adsorption was estimated by subtracting the background ion desorption.

### Statistical analyses

ANOVA and regression analyses were performed in SPSS. Regression analyses were first performed in Excel and then verified in SPSS. Specifically, regression analyses were used to assess the correlations between pH and sorption, displacement and sorption, and actual versus modeled sorption (using Freundlich and Langmuir models). A two-way ANOVA was used to assess the effects of biochar feedstock and pyrolysis temperature on sorption of NH_4_^+^ and NO_3_^−^ to the biochars.

## Results

### Elemental composition of biochars

Major elemental composition of the six acid-washed biochars varied with respect to both feedstock and pyrolysis temperature (Table 1). Consistent with the literature, red oak biochar had higher %C, and lower %N and %S compared to the corn stover biochar produced at the same temperature^36–38^. Red oak biochar also had higher molar C:N ratios and lower H:C ratios compared with corn stover biochar. This agrees with previous observations of higher C content with increasing pyrolysis temperatures, which is accompanied by decreasing H and %S content^36–38^. Because the analyzed biochar had been acid-washed, the %C represents total organic C of the biochar, and the mass not accounted for by %C, N, H or S would be composed of primarily SiO_2_, Fe, Al, and organic O^30^. This remaining percent mass was higher for corn stover than red oak biochar, and decreased with increasing pyrolysis temperatures. A decrease in O content with increasing pyrolysis temperature is consistent with increased aromatization, and higher Si in corn stover biochar is consistent with higher Si content of corn stover compared to wood^30,39,40^.

**Table 1:** Total C, N, H and S concentrations (weight %) of the six acid-washed biochars, and their C:N and H:C ratios (molar) (±s.d., n = 3).

biochar	%C	%N	%H	%S	C:N	H:C
RO4s	79.1 ± 1.1	0.22 ± 0.02	4.07 ± 0.03	0.08 ± 0.05	431 ± 33	0.617 ± 0.005
RO5s	86.1 ± 0.8	0.25 ± 0.01	3.26 ± 0.03	0.02 ± 0.01	409 ± 22	0.454 ± 0.004
RO6s	91.6 ± 0.5	0.3 ± 0.05	2.49 ± 0.03	0.01 ± 0.01	370 ± 68	0.326 ± 0.003
CS4s	73.2 ± 0.5	1.2 ± 0.01	4.14 ± 0.04	0.1 ± 0.06	71 ± 1	0.678 ± 0.003
CS5s	78.7 ± 0.2	1.15 ± 0.01	3.13 ± 0.03	0.06 ± 0.01	78 ± 3	0.476 ± 0.003
CS6s	82.2 ± 0.7	1.06 ± 0.05	2.31 ± 0.03	0.05 ± 0.01	90 ± 4	0.338 ± 0.003

### Sorption of NH_4_^+^ and NO_3_^−^ to biochar from acidic to neutral solutions

**Figure 1:**
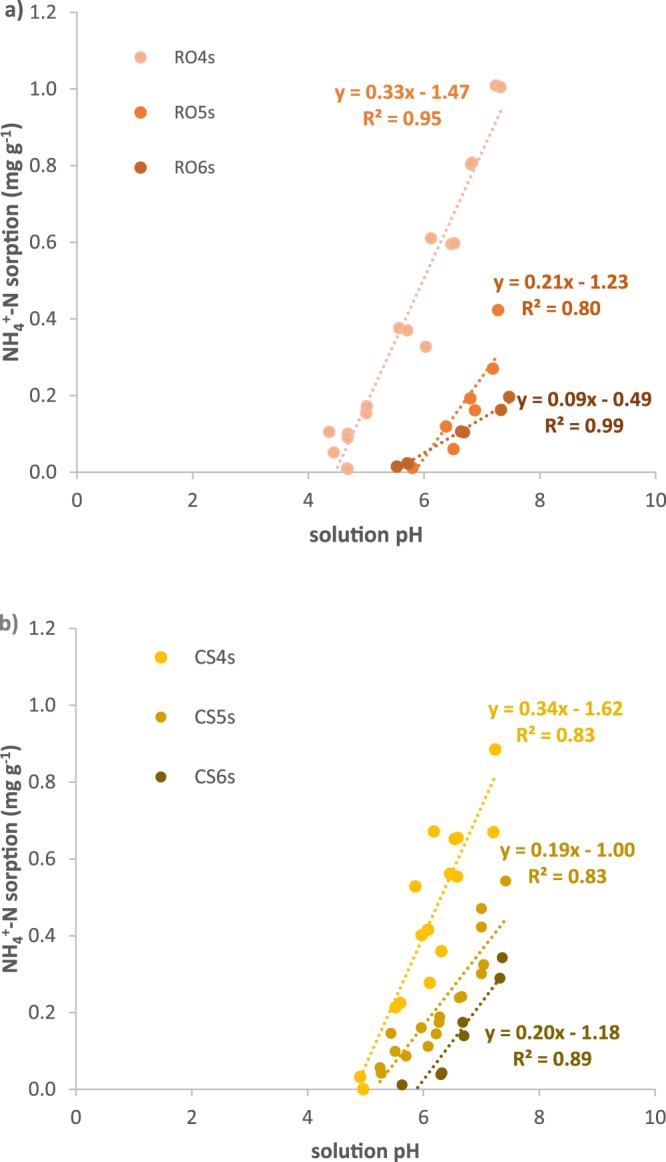
Sorption of NH_4_^+^ to acid-washed (**a**) red oak and (**b**) corn stover biochars from 10 mg N L^−1^ solutions adjusted to various pHs using Ca(OH)_2_.

For both feedstocks, NH_4_^+^ sorption significantly increased with increasing solution pH and with decreasing pyrolysis temperature (Fig. 1). A linear model provided the best fit for explaining variation in NH_4_^+^ sorption with respect to pH (*r*^2^ = 0.80–0.99) (due to acceptable fits [r^2^ ≥ 0.7] for all curves using both linear and exponential models, we defer discussion regarding the significance of best fit curve shapes to future studies). Without the addition of Ca(OH)_2_ to increase the pH to >4, NH_4_^+^ sorption was negligible for all biochars (<0.02 mg g^−1^). At pH 7, NH_4_^+^ sorption decreased with increasing pyrolysis temperature for both feedstocks: the 400, 500 and 600 °C biochars sorbed 0.74–0.84, 0.25–0.42, and 0.14–0.23 mg NH_4_^+^-N g^−1^, respectively. Within each feedstock, biochars produced at different temperatures had significantly different NH_4_^+^ sorption rates (p < 0.01) – except CS4s and CS5s, which did not have significantly different sorption rates (p > 0.05). The effect of feedstock on NH_4_^+^ sorption at pH 7 was not consistent; rather, red oak biochar showed a wider range of sorption (0.14 to 0.84 mg NH_4_^+^-N g^−1^) compared with corn stover biochar (0.23 to 0.74 mg NH_4_^+^-N g^−1^) as biochar pyrolysis temperature increased from 400–600 °C (RO6s < CS6s < RO5s ≤ CS5s < CS4s < RO4s). This effect of feedstock was significant among biochars produced at 400 °C and 500 °C (p < 0.05), but not at 600 °C (p > 0.05). Thus, controlling for pH, both effects of pyrolysis temperature (0.17–0.42 mg NO_3_^−^-N g^−1^ difference per 100 °C change in pyrolysis temperature) and feedstock (0.004–0.45 mg NO_3_^−^-N g^−1^ difference at a given temperature) were observed.

**Figure 2:**
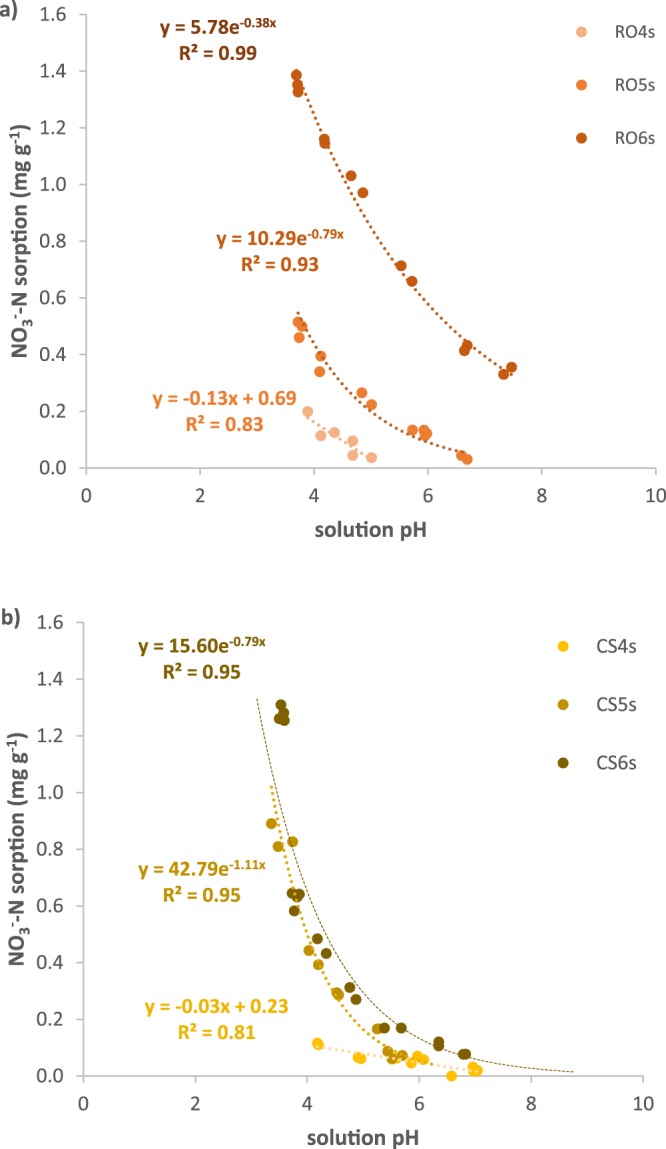
Sorption of NO_3_^−^ to acid-washed (**a**) red oak and (**b**) corn stover biochars from 10 mg N L^−1^ solutions adjusted to various pHs using Ca(OH)_2_.

Opposite from NH_4_^+^, NO_3_^−^ sorption increased with decreasing solution pH and increasing pyrolysis temperature (Fig. 2). Variation in NO_3_^−^ sorption with respect to pH was best fit with exponential models for 500–600 °C biochars (*r*^2^ = 0.93–0.99) and linear models for 400 °C biochars (*r*^2^ = 0.81–0.83). Sorption of NO_3_^−^ was highest without addition of Ca(OH)_2_; for all but one biochar (RO6s), sorption declined to < 0.05 mg NO_3_^−^-N g^−1^ when sufficient Ca(OH)_2_ was added to achieve a pH of 7–7.5. The main effect of *temperature* and the *feedstock* × *temperature* interaction were significant (p < 0.05), but the main effect of *feedstock* was not significant (p > 0.05). At pH 3.7, NO_3_^−^ sorption statistically increased with increasing pyrolysis temperature for both feedstocks, with the 400, 500 and 600 °C biochar removing 0.15–0.20, 0.49–0.85, and 1.35–1.49 mg NO_3_^−^-N g^−1^, respectively. For all pyrolysis temperatures, NO_3_^−^ sorption to corn stover biochar declined more rapidly with increasing pH than NO_3_^−^ sorption to red oak biochar. Red oak biochar was equal to or superior to corn stover biochar for NO_3_^−^ sorption, and sorption increased in the order CS4s ≤ RO4s < RO5s < CS5s < RO6s ≤ CS6s. The effect of feedstock on NO_3_^−^ sorption was significant for biochars produced at 400 °C and 600 °C (p < 0.01), but not for biochars produced at 500 °C (p > 0.05). Thus, controlling for pH, both effects of temperature (0.6–1 mg NO_3_^−^-N g^−1^ difference per 100 °C change in pyrolysis temperature) and feedstock (0.05–0.36 mg NO_3_^−^-N g^−1^ difference at a given temperature) were observed.

Thus, NH_4_^+^ and NO_3_^−^ sorption exhibits opposite trends with respect to biochar pyrolysis temperature and pH: with increasing pyrolysis temperature and decreasing pH, NH_4_^+^ sorption decreased whereas NO_3_^−^ sorption increased. The main effect of feedstock itself was not significant (p > 0.05). However, *feedstock*.× *temperature* interactions are evident for sorption of both N-species at select pHs; for example, NO_3_^−^ sorption was slightly higher for red oak biochar than corn stover biochar at 600 °C and slightly lower at 500 °C (no Ca(OH)_2_ added) (p < 0.05). The effect of feedstock was largest for 500 °C biochars (RO5s and CS5s), which had NH_4_^+^ and NO_3_^−^ sorption differing by 0.21 and 0.36 mg N g^−1^, respectively. Therefore, NH_4_^+^ and NO_3_^−^ sorption appeared to be primarily a function of solution pH and biochar pyrolysis temperature, with a small but significant *feedstock *× *temperature* interaction effect (p < 0.05).

### Sorption isotherms for corn stover biochars

**Figure 3:**
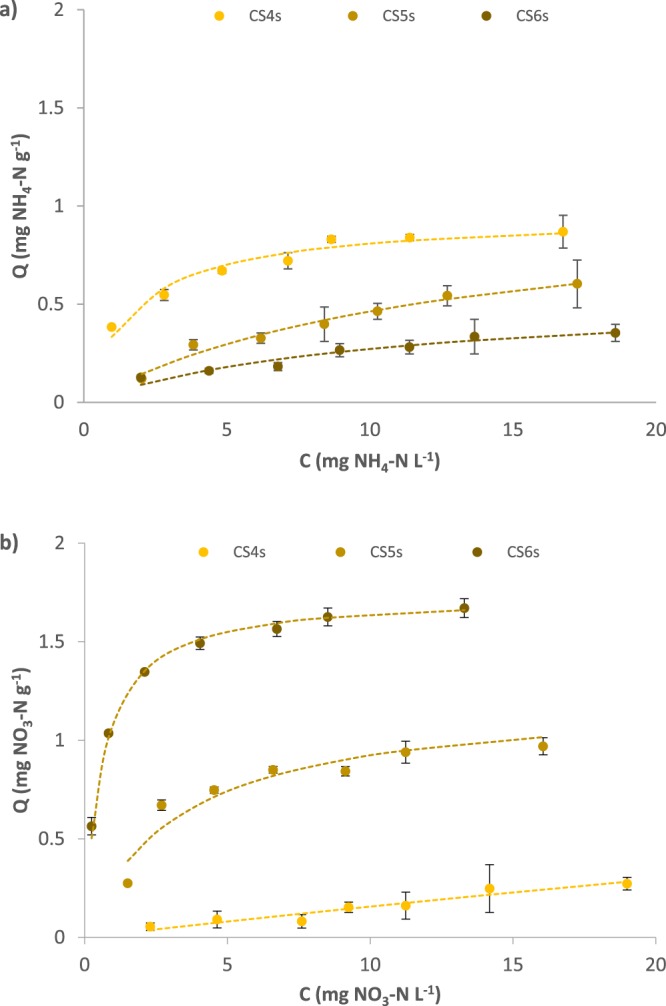
Sorption isotherms of (**a**) NH_4_^+^ (pH 7) and (**b**) NO_3_^−^ (pH 3.7) for acid-washed corn stover biochars, fit with the Langmuir model (C = final solution concentration; Q = final sorbed concentration). Error bars indicate ± s.d. (n = 3).

Sorption of NH_4_^+^ and NO_3_^−^ to corn stover biochar increased from 2.5–20 mg N L^−1^ with increasing equilibrating solution concentration (Fig. 3) and were better fit using Langmuir isotherm models (r^2^ = 0.89 to 0.99) compared with Freundlich isotherm models (r^2^ = 0.78 to 0.95) (Tables S1 and S2). Consistent with the pH curves, as pyrolysis temperature increased, NH_4_^+^ sorption (at pH ~7) decreased and NO_3_^−^ sorption (at pH ~3.7) increased. For biochars with lower sorption capacities, often <20% of the initial N was sorbed; therefore, we interpret curves showing low sorption efficiency with caution (NH_4_^+^ sorption to CS5s and CS6s, and NO_3_^−^ sorption to CS4s) and emphasize sorption curves with higher efficiencies in our interpretation of sorption mechanisms. Each Langmuir isotherm yielded equal or greater r^2^ values than respective Freundlich isotherms generated for the same biochar, including those with the highest sorption rates (CS4s for NH_4_^+^ and CS6s for NO_3_^−^) (r^2^ = 0.96 to 0.99 for Langmuir; r^2^ = 0.90 to 0.94 Freundlich). Sorption capacities (S_max_) and binding energies (K) for NH_4_^+^ did not show a consistent trend with pyrolysis temperature (although both S_max_ and K were higher for CS4s than CS6s); however, for NO_3_^−^, K increased consistently with pyrolysis temperature. Thus, the sorption isotherm results were consistent with an effect of biochar pyrolysis temperature as well as an ion exchange sorption mechanism for both sorbates.

### Ion displacement from corn stover biochar

**Figure 4:**
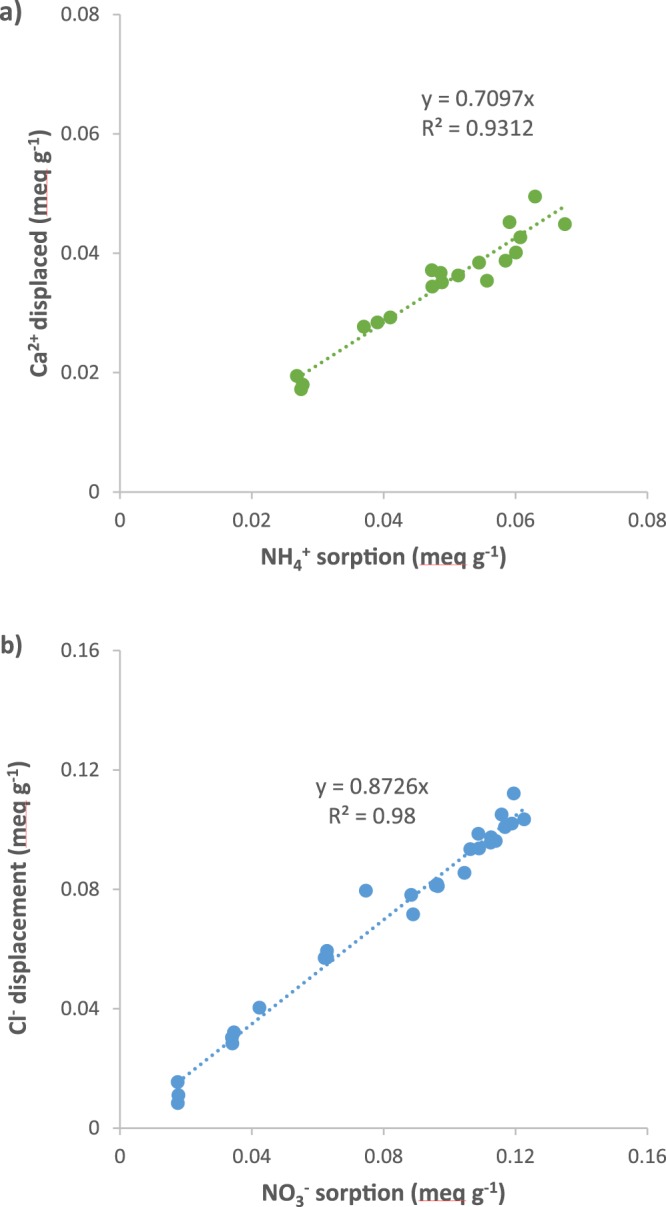
Displacement of (**a**) Ca^2+^ during NH_4_^+^ sorption to CS4s and (**b**) Cl^−^ during NO_3_^−^ sorption to CS6s.

Quantities of Ca^2+^ and Cl^−^ displaced from CS4s and CS6s biochars were positively correlated (p < 0.001) with quantities of NH_4_^+^ and NO_3_^−^ sorbed, respectively (Fig. 4) (see Fig. 3 for corresponding sorption isotherms). The ratio of and Ca^2+^ displaced to NH_4_^+^ sorbed (meq g^−1^: meq g^−1^) was 0.71 (r^2^ = 0.93) (Fig. 4a), and the ratio of Cl^−^, displaced to NO_3_^−^ sorbed was 0.87 (r^2^ = 0.98) (Fig. 4b). Thus, there was a linear increase in counter-ion desorption for both N species, but the relationship between sorption and displacement was not stoichiometric (1:1). This observed displacement of counter-ions supports ion exchange as the dominant mechanism for both NH_4_^+^ and NO_3_^−^, but the displacement ratio of less than 1:1 implicates a potentially more complex (multi-mechanism) sorption reaction.

## Discussion

Previous studies investigating NH_4_^+^ and NO_3_^−^ sorption on biochars have yielded conflicting results, which may have arisen due to the confounding influences of native alkalis and soluble salts in biochars. In order to disentangle effects of biochar production parameters and solution pH on NH_4_^+^ and NO_3_^−^ sorption and to elucidate sorption mechanisms, we analyzed NH_4_^+^ and NO_3_^−^ sorption to six acid-washed and CaCl_2_-saturated biochars at acidic to neutral solution pHs, and quantified counter ion displacement during NH_4_^+^ and NO_3_^−^ sorption. At the time of writing, this is the first NH_4_^+^ and NO_3_^−^ sorption study to control for both solution pH and competitive ions. Results confirmed our hypotheses that biochar feedstock and pyrolysis temperature as well as solution pH influence NH_4_^+^ and NO_3_^−^ sorption, and sorption occurs primarily through ion exchange. These hypotheses were supported by: (1) decreasing NH_4_^+^ and increasing NO_3_^−^ sorption with increasing biochar pyrolysis temperature, (2) differences in sorption between red oak and corn stover biochars at most pyrolysis temperatures, (3) increasing NH_4_^+^ and decreasing NO_3_^−^ sorption with increasing solution pH, (4) good fit of the Langmuir isotherm, and (5) strong positive correlations between Ca^2+^ and Cl^−^ displacement and NH_4_^+^ and NO_3_^−^ sorption, respectively.

In general, there are 4 major classes of mechanisms for describing ion sorption onto biochars: surface precipitation (salt precipitation), adsorbed chemically (chemical reaction with surface functional groups), entrapped in the solution present in interior pores (physical absorption), or adsorbed electrostatically (cation or anion exchange to charged functional groups on biochar surfaces)^41,42^. Greater sorption capacity for NH_4_^+^ by lower temperature biochars has been attributed to higher cation exchange capacities (CEC) – arising from greater concentrations of carboxylate groups, which provide negatively charged surface adsorption sites^23–29^. Despite inconsistent controls on pH and competing cations in the literature, the increasing NH_4_^+^ sorption with increasing pH observed here is consistent with previous studies^23–25^. Recall that, under acidic conditions, ammoniacal-N exists mainly as NH_4_^+^; however, at pH significantly greater than 7 the non-ionic form (NH_3_) becomes the dominant species^43^. In addition, at lower pH values the ionic form NH_4_^+^ would compete with the increasing H^+^ ion concentration for cation exchange sites. This does pose serious hurdles in interpreting the pH dependency, since the mode of sorption can fundamentally change. In our experiment, the pH was limited to solely the acidic to neutral region where the mechanism was assumed to involve solely the ionic NH_4_^+^ species. The solution pH controls important factors of surface charge of the sorbent, degree of ionization/speciation of the sorbate^44^. For example, carboxylic acid functional groups (pK_a_ < 6.4) and lactones or lactols (6.4 < pK_a_ < 10.3) on biochar surfaces would become deprotonated as pH increases, increasing the concentration of negatively charged cation exchange sites^30,33,45^. Furthermore, functional group concentrations with pK_a_ < 6.4 for acid-washed biochars produced using the same pyrolyzer and at similar temperatures to those analyzed here – likely to make the largest contribution to sorption within the 3.7–7 pH range used here – were previously shown to be higher for biochars produced at lower temperatures^30,33^. Indeed, this finding is in agreement with the aforementioned studies reporting increasing carboxylic acid concentrations and/or CEC’s with decreasing pyrolysis temperature^23–29,46–49^. A cation exchange mechanism is further supported by the displacement of Ca^2+^ in proportion with NH_4_^+^ sorption (Fig. 4a). In this case, Ca^2+^ was displaced by NH_4_^+^ sorption at a rate of 0.71:1; this rate of <1:1 may arise from a secondary, less-dominant sorption mechanism co-occurring with cation exchange (it should be noted that Ca^2+^ and NH_4_^+^ were the only cations analyzed, and hence trace quantities of cations left over from acid washing may have competed for a small percentage of sorption sites). The comparable fits of the Langmuir and Freundlich isotherms to NH_4_^+^ sorption curves for corn stover biochars further supports the possibility of multiple mechanisms. The slightly better fit of the Langmuir isotherms may be the product of a dominant electrostatic adsorption mechanism alongside another mechanism (Fig. 3a). Thus, the results of this study confirm that adsorption of NH_4_^+^ to biochar occurs primarily through cation exchange on negatively charged biochar functional groups, and is consequently maximized with lower pyrolysis temperatures, lower cation competition, and higher solution pH.

The NO_3_^−^ sorption and concurrent Cl^−^ displacement data measured here confirm that NO_3_^−^ sorption increases with increasing biochar pyrolysis temperature and decreasing pH, likely reflecting an anion exchange sorption mechanism. Nitrate sorption measured at pH 3.7 ± 0.2 for corn stover biochar pyrolyzed at 500 °C (0.85 mg N g^−1^) falls within the range reported by Gai *et al*.^26^ and Chintala *et al*.^4^ (0.45–2.5 mg N g^−1^) despite inconsistent controls on pH and competing ions in previous studies. The pH curves, Langmuir isotherms and Cl^−^ displacement data reported here together suggest that surface moieties sorb NO_3_^−^ through an anion exchange mechanism. Increased anion exchange capacity (AEC) of biochar with increasing pyrolysis temperature and decreasing pH has been reported by Lawrinenko *et al*.^50^. Lawrinenko *et al*. hypothesized that biochar AEC resulted from oxonium and pyridinum groups. Oxonium groups provide pH-independent anion exchange sites; however, at high pHs OH^−^ groups would compete with NO_3_^−^ for binding sites. Furthermore, non-bridging oxonium groups are subject to nucleophilic attack by OH^−^, thereby decreasing AEC and NO_3_^−^ sorption. Competition with other anions (PO_4_^3−^ and SO_4_^2−^) reported elsewhere^4^ is in agreement with this anion exchange mechanism. Here Cl^−^ was displaced by NO_3_^−^ sorption at a rate of 0.87:1, perhaps reflecting a secondary mechanism occurring alongside anion exchange. It should be noted that only Cl^−^ and NO_3_^−^ were analyzed, and hence trace amounts of other anions remaining after acid-washing may have competed for sorption sites. Given the saturation with Ca^2+^, some sorption via cation bridging may have occurred as well. Alternatively, small quantities of NO_3_^−^ could have become entrapped in or precipitated in micro- or nanopores^15,41^. Nevertheless, the results are consistent with adsorption of NO_3_^−^ to biochar through an anion exchange mechanism, and indicate that NO_3_^−^ sorption is consequently maximized with higher pyrolysis temperatures, reduced anion competition, and lower solution pH.

The contrasting responses of NH_4_^+^ and NO_3_^−^ sorption to pyrolysis temperature and solution pH have implications for the use of biochar for N pollution mitigation. A trade-off between NH_4_^+^ and NO_3_^−^ sorption capacity is apparent. In other words, a single biochar cannot be simultaneously optimized for sorption of both N species. Furthermore, due to the pH sensitivity of N sorption, NH_4_^+^ and NO_3_^−^ sorption cannot be simultaneously optimized at any given pH. These trade-offs present a challenge, but also an opportunity to tailor biochars to specific applications. For example, low temperature biochars might be produced for enhancing NH_4_^+^ sorption, and their performance would be optimal under slightly acidic to neutral conditions (pH 6–7.5). Use in more alkaline systems (pH > 7.5) may be effective, but the alkaline environment promotes ammonia volatilization and consequential loss of sorbed NH_4_^+^. By contrast, a high temperature biochar would be desirable for retention of NO_3_^−^, and its performance would be optimized in acidic environments (pH < 6). In this study, feedstock effects were relatively small in comparison to pyrolysis temperature and pH effects, but analysis of biochars produced from a more diverse suite of feedstocks – including manures and nut shells, for example – may yet reveal significant feedstock trade-offs. Moreover, there is evidence that competitive ions in solution will influence overall sorption capacity, as observed in the low affinity of the sorbate (nitrate or ammonium; Freundlich n < 1) and in the displacement of counter-ions (Fig. 4, Tables S1 and S2). Consequently, biochar pyrolysis temperature, biochar alkalinity, soluble ash composition, and soil pH may explain a large proportion of variability in the literature examining biochar impacts on the N cycle and crop production^6,10,51^.

The sensitivity of biochar NH_4_^+^ and NO_3_^−^ sorption to pH and competitive ions necessitates a closer examination of their roles in studies examining biochar impacts on N cycling. In N sorption studies, meaningful direct comparisons cannot be made among biochars with varying pH and ash contents. Both alkalis and salts will influence N sorption in the short-term, and their influence will likely diminish over time in applications where these soluble biochar components are leached by water, rendering short-term sorption studies with untreated biochars misrepresentative of actual field conditions. Therefore, we strongly recommend accounting for both soluble alkalis and salts when comparing ion sorption capacities of biochars, as appropriate to the hypothesis of interest. Acid-washing of biochars provides a valuable approach to generating more meaningful comparisons of behaviors and related biochar properties among diverse biochars in basic research experiments. Without such pre-treatments, biochar alkalis and soluble salt concentrations – both shown to increase with increasing pyrolysis temperature^30,52^ – may produce systematic underestimates in measured sorptive capacities, and furthermore diminish the apparent effect of biochar pyrolysis temperature. On the other hand, the efficacy of biochars in complex chemical environments as encountered in soils, storm water, and natural effluents will undoubtedly depend on how sorbates of interest compete with other ions in solution at various pHs. Overall, more applied research is needed to demonstrate effective use of biochars for diverse real-world purposes, and reactive biochar components – especially soluble salts and alkalis – must be considered at all stages of biochar research and development.

To conclude, this study demonstrates that NH_4_^+^ and NO_3_^−^ sorption vary as opposite functions of biochar pyrolysis temperature and solution pH, and are also influenced by biochar feedstock and competing ion concentration. Results were consistent with a predominant ion exchange mechanism of sorption, indicating that solution pH and ion concentration must be controlled to enable accurate comparisons across different studies. Although these findings present challenges to experimental design, data interpretation, and the practical use of biochar as a whole, they also represent an opportunity to engineer specialized biochars and optimize their use for diverse applications.

## Electronic supplementary material

Supplemental Information
