# Supplementary material for: Sorption of ammonium and nitrate to biochars is electrostatic and pH-dependent
Source: Sci Rep. 2018 Dec 4;8:17627. doi: 10.1038/s41598-018-35534-w (PMC6279760; doi:10.1038/s41598-018-35534-w)
Supplement: Supplementary file 1 — Supplemental Information [file 41598_2018_35534_MOESM1_ESM.docx]

***Supplemental Information***

**Sorption of ammonium and nitrate to biochars is electrostatic and pH-dependent**

Rivka B. Fidel, David A. Laird and Kurt Spokas

| **Table S1** Freundlich and Langmuir sorption isotherm fit parameters for sorption of NH_4_^+^ to corn stover biochars | | | | | | |
| --- | --- | --- | --- | --- | --- | --- |
|  | Freundlich | | | Langmuir | | |
| biochar | K_f_ | 1/n | r^2^ | S_max_ | K | r^2^ |
| CS4s | 0.418 | 0.280 | 0.955 | 0.954 | 0.554 | 0.959 |
| CS5s | 0.110 | 0.610 | 0.966 | 1.048 | 0.079 | 0.975 |
| CS6s | 0.077 | 0.534 | 0.949 | 0.556 | 0.096 | 0.929 |

| **Table S2** Freundlich and Langmuir sorption isotherm fit parameters for sorption of NO_3_^-^ to corn stover biochars | | | | | | |
| --- | --- | --- | --- | --- | --- | --- |
|  | Freundlich | | | Langmuir | | |
| biochar | K_f_ | 1/n | r^2^ | S_max_ | K | r^2^ |
| CS4s | 0.019 | 0.922 | 0.92 | 2.72 | 0.006 | 0.92 |
| CS5s | 0.407 | 0.339 | 0.81 | 1.16 | 0.355 | 0.90 |
| CS6s | 1.032 | 0.213 | 0.91 | 1.71 | 1.879 | 1.00 |

**Figure S1** Freundlich isotherm for sorption of ammonium to corn stover biochars

**Figure S2** Freundlich isotherm for sorption of nitrate to corn stover biochars
